# LEFTY2/endometrial bleeding-associated factor up-regulates Na^+^ Coupled Glucose Transporter SGLT1 expression and Glycogen Accumulation in Endometrial Cancer Cells

**DOI:** 10.1371/journal.pone.0230044

**Published:** 2020-04-01

**Authors:** Ni Zeng, Toshiyuki Okumura, Md Alauddin, Shayan Khozooei, Janet Rajaxavier, Shaqiu Zhang, Yogesh Singh, Bing Shi, Sara Y. Brucker, Diethelm Wallwiener, Satoru Takeda, Florian Lang, Madhuri S. Salker

**Affiliations:** 1 State Key Laboratory of Oral Diseases & National Clinical Research Center for Oral Diseases & West China Hospital of Stomatology, Sichuan University, Chengdu, China; 2 Department of Cleft Lip and Palate Surgery, West China Hospital of Stomatology, Sichuan University, Chengdu, China; 3 Women’s Health Research Institute, Department of Obstetrics and Gynecology, University of Tübingen, Tübingen, Germany; 4 Department of Obstetrics and Gynecology, Juntendo University School of Medicine, Tokyo, Japan; 5 Institute of Preventive Veterinary Medicine, Sichuan Agricultural University, Wenjiang, Chengdu City, Sichuan, China; 6 Institute for Medical Genetics and Applied Genomics, University of Tübingen, Tübingen, Germany; 7 Department of Internal Medicine III, University of Tübingen, Tübingen, Germany; Yonsei University College of Medicine, REPUBLIC OF KOREA

## Abstract

LEFTY2 (endometrial bleeding associated factor; EBAF or LEFTYA), a cytokine released shortly before menstrual bleeding, is a negative regulator of cell proliferation and tumour growth. LEFTY2 down-regulates Na^**+**^/H^**+**^ exchanger activity with subsequent inhibition of glycolytic flux and lactate production in endometrial cancer cells. Glucose can be utilized not only for glycolysis but also for glycogen formation. Both glycolysis and glycogen formation require cellular glucose uptake which could be accomplished by the Na^**+**^ coupled glucose transporter-1 (SGLT1; SLC5A1). The present study therefore explored whether LEFTY2 modifies endometrial SGLT1 expression and activity as well as glycogen formation. Ishikawa and HEC1a cells were exposed to LEFTY2, *SGLT1* and *glycogen synthase* (*GYS1*) transcript levels determined by qRT-PCR. SGLT1, GYS1 and phospho-GYS1 protein abundance was quantified by western blotting, cellular glucose uptake from 2-(*N*-(7-Nitrobenz-2-oxa-1,3-diazol-4-yl)Amino)-2-Deoxyglucose (2-NBDG) uptake, and cellular glycogen content utilizing an enzymatic assay and subsequent colorimetry. As a result, a 48-hour treatment with LEFTY2 significantly increased *SGLT1* and *GYS1* transcript levels as well as SGLT1 and GYS1 protein abundance in both Ishikawa and HEC1a cells. 2-NBDG uptake and cellular glycogen content were upregulated significantly in Ishikawa (type 1) but not in type 2 endometrial HEC1a cells, although there was a tendency of increased 2-NBDG uptake. Further, none of the effects were seen in human benign endometrial cells (HESCs). Interestingly, in both Ishikawa and HEC1a cells, a co-treatment with TGF-β reduced SGLT1, GYS and phospho-GYS protein levels, and thus reduced glycogen levels and again HEC1a cells had no significant change. In conclusion, LEFTY2 up-regulates expression and activity of the Na^**+**^ coupled glucose transporter SGLT1 and glycogen synthase GYS1 in a cell line specific manner. We further show the treatment with LEFTY2 fosters cellular glucose uptake and glycogen formation and TGF-β can negate this effect in endometrial cancer cells.

## Introduction

LEFTY2 (endometrial bleeding associated factor; EBAF or LEFTYA) is a member of the transforming growth factor beta (TGF-β) superfamily. LEFTY2 is produced as a precursor protein that is cleaved, leading to release of the C-terminus monomeric active proteins [1]. Unlike other TGF-β family members, LEFTY2 does not function *via* receptor-mediated SMAD-dependent signaling, but rather by antagonizing the signaling of TGF- β and Nodal [2]. In brief, activin, belonging to TGF-β superfamily, binds to type II ActRII receptor, causing the phosphorylation and activation of the type I activin-like kinase 4 (ALK4; TGFR) receptor [3]. Activated ALK4 phosphorylates in turn SMAD proteins (SMAD2 and SMAD3) [4] forming complexes with SMAD4. The activated complexes translocate into the nucleus and affect TGF-β specific genes [3]. LEFTY2 can antagonize the signaling pathway by interacting with ActRII, thus blocking phosphorylation of SMAD and inhibiting downstream factors [3]. It is now well established that tumorigenesis is associated with development of resistance to TGF-β signaling, and for this reason, it is thought that TGF-β signaling acts as a potent tumor suppressor [5]. Since the normal function of the TGF-β signaling pathway is suppression of cellular proliferation and transformation, it could be proposed that the action of LEFTY2 could be a potential oncoprotein by counteracting TGF-β-mediated signaling. Further, LEFTY2 is highly enriched in embryonic stem cells and participates in the regulation of ‘stemness’ and embryonic differentiation [6–9]. This expression has been shown to re-appear in cancers, such as breast and melanoma [10].

Tumors reprogram nutrient pathways to meet the high bio-energetic demands of malignant cells [11, 12]. These reprogrammed activities are now acknowledged as the hallmarks of cancer [12, 13]. The reprogrammed metabolic pathway in cancer is known as aerobic glycolysis, a phenomenon known as the “Warburg effect” [11]. In the 1920s, Nobel Laureate Otto Warburg described that tumor slices and malignant ascites (presence of malignant cells in the peritoneal cavity) constitutively take up glucose and produce lactate irrespective of oxygen availability [14]. Glycolysis is a physiological response to hypoxia in normal tissues. Glycolysis fuels a substantial portion of ATP production in cancer cells [15–21] and is decisive for energy production particularly during ischemia [22]. Previously, LEFTY2 was shown to be an inhibitor of cell proliferation and is capable of stimulating apoptosis [23–26], thereby counteracting tumor growth [27–30]. LEFTY2 is partially effective by down-regulating Na^+^/H^+^ exchanger 1 (NHE-1), leading to a decrease of glycolytic flux (the rate at which molecules proceed through the glycolytic pathway) in endometrial cancer cells [31]. Glycolytic flux requires the maintenance of alkaline cytosolic pH since the rate-limiting enzymes of glycolysis are highly pH-sensitive and inhibited by cytosolic acidification [32]. In tumor cells, an alkaline cytosolic pH is accomplished by several transporters including the Na^+^/H^+^ exchangers (NHE1-9) [15, 33], Na^+^ coupled bicarbonate co-transporters [33] and lactate or mono-carboxylate transporters [15, 33] extruding both, lactate and H^+^ ions [34].

Maintenance of glycolytic flux critically depends on the supply of glucose. Normally, the delivery of glucose is partially accomplished by the passive glucose carriers of the GLUT family [35, 36]. Glucose may in addition, be taken up by the Na^+^-Glucose co-transporter (SGLT) family. The SGLTs mediate secondary active transport driven by the Na^+^ gradient across the cell membrane [37, 38]. The two members of this protein family, SGLT1 and SGLT2, accomplish the concentrative cellular uptake of glucose across the apical cell membrane of epithelial cells [37].

SGLT1 expression is, however, not limited to healthy epithelial tissues, but has been identified in several tumor cells, which have an increased substrate demand and utilize mainly glucose for energy production [37, 39, 40]. SGLT1 allows cellular glucose uptake against a glucose concentration gradient and is particularly important for cell survival during ischemia and excessive glucose utilization [41–43]. Recently, it has been shown that SGLT1 is present in the endometrium and plays a decisive role in pregnancy outcome in both humans and mice [44]. We therefore sought to investigate the relationship of LEFTY2 and SGLT1 in endometrial cancer cells.

The present study investigated whether LEFTY2 affects the Na^+^ coupled glucose transporter SGLT1 in human endometrial (Ishikawa and HEC1a) cancer cells. Surprisingly, LEFTY2 stimulated SGLT1 expression and activity. Further studies revealed that LEFTY2 fosters the incorporation of accumulated glucose into glycogen. None of these effects were seen in healthy endometrial cells. Interestingly, co-treatment with TGF-β negated the LEFTY induced effect.

## Materials and methods

### Cell culture

Ishikawa cells, a well differentiated endometrial carcinoma cell line (#ECACC 99040201) [45], Hec1A (type 2 endometrial adenocarcinoma; #HTB-112 purchased from ATCC) and benign human endometrial cells (#T0533; HESCs purchased from Applied Biological Materials Inc., Richmond, Canada) were routinely grown in monolayers using 75 cm^2^ culture flasks maintained at 37°C in a humid atmosphere containing 5% (v/v) carbon dioxide (CO_2_) using a Heracell incubator. Cells were maintained in DMEM: F12 phenol free supplemented with 10% (v/v) FBS, 1% antibiotic/antimycotic solution and 1% L-Glutamine (Invitrogen, Germany), which was changed every other day and passaged when near confluent (1–2 times per week depending on growth rate). All work was carried out in a Class I safety cabinet. Cells were treated with LEFTY2 (25 ng/ml; 746-LF-025/CF; recombinant human: R&D Systems, Germany) or with TGF-β (10 ng/ml,14-8342-80, ebioscience, USA) in DMEM: F12 phenol free supplemented with 2% (v/v) Fetal Bovine Serum, 1% antibiotic/antimycotic solution and 1% L-Glutamine for 48 hours (all from Invitrogen, Germany). No Institutional review board approval was required for this study.

### Messenger RNA (mRNA) extraction and quantitative real-time PCR (qRT-PCR)

RNase ZAP, RNase-free plastic-ware and DEPC-treated water were used to minimize degradation of mRNA. Total mRNA was extracted from cells cultured in 6-well plates by direct lysis using the miRNeasy® Mini Kit (Qiagen, Germany) according to the manufacturer’s protocol. The mRNA concentration was determined using a Nanodrop (Eppendorf μCuvette^®^ G1.0, Microvolume measuring cell for Eppendorf BioPhotometer^®^ and BioSpectrometer^®^, Eppendorf, Germany) and the A260/ A280 ratio of 1.9–2.1 was used as a threshold. Samples with lower ratios indicating contamination were discarded. Samples were diluted to 1 μg/ μl with DEPC- water and stored at -80°C.

One μg RNA was reverse transcribed by using the ThermoScientific Maxima^TM^ H Minus cDNA Synthesis Master Mix with dsDNase (Invitrogen). The resulting cDNA was diluted and used as a template for subsequent PCR reactions. Primers were designed using the NCBI primer blast software. Ribosomal L19 (housekeeping) was used to normalize for discrepancies in input cDNA. Gene expression was quantified using the PowerUp^TM^ SYBER^®^ Green Master Mix (Invitrogen) and performed using the QuantStudio 3 Real-Time PCR System (Invitrogen). In each PCR reaction a non-template control (NTC) reaction (where cDNA is substituted with DNase/RNase free water) and reverse transcriptase (-RT) controls were included. The PCR products were not detected in NTC or RT control reactions. Primer sequences: *hSGLT1* forward (5'-3'): AGAGGGGAACAGACAACACA & reverse (5'-3'): ACCAAAACCAGGGCATTCCA, *hGYS1* forward (5’-3’): AGGGCTGCAAGGTGTATTTC & reverse (5’-3’): ACTCCGATGTTGCAGGTATC, housekeeping *hL19* forward (5'-3'): GCAGCCGGCGCAAA & reverse (5'-3'): GCGGAAGGGTACAGCCAAT. Expression levels were calculated using the ^ΔΔ^C_T_ method. The values are provided as arbitrary values (a.u.). All measurements were performed in triplicate. Melting curve analysis and agarose gel electrophoresis was also performed to confirm amplification specificity.

### Western blotting

Whole cell protein extracts were harvested from 6-well plates using Laemmli buffer following a PBS wash (1ml/well). Cell scrapers were used to collect the lysates which were then pipetted into 1.0 ml tubes and heated for 10 min at 95°C [46]. Proteins were resolved on 10% sodium dodecyl sulfate–polyacrylamide (SDS) gels using the Invitrogen XCell SureLock® Mini-Cell apparatus. 5 μl protein ladder (Biozym, Germany) was added to the first lane. Gels were run at 125 V for up to 2 hours until the dye front had migrated to the base of the gel, at which time the cassettes were opened and the gels used for transfer. The gels were transferred onto a PVDF membrane (Amersham Biosciences, Germany), activated in methanol, using a wet-transfer blotting method. The transfer was performed at 230 mA for 2 hours in a box of ice. The PVDF membrane was then air-dried and reactivated in methanol before being incubated with 5% non-fat dry milk in Tris-buffered saline with 1% Tween (TBS-T) (TBS; 130 mmol/L NaCl, 20 mmol/L Tris, pH 7.6 and 1% Tween) for 1h (RTP). This procedure aimed to prevent nonspecific binding. The membrane was then washed (once in TBS-T for 5 minutes and three times in TBS-T for 15 minutes) before incubation with the primary antibody. SGLT1, phosphorylated GYS1 (Ser641), and GYS1 were identified by primary antibodies against human SGLT1 (1:1000, #5042, Cell Signaling, Netherlands), human phosphorylated GYS1 (Ser641) (1:1000, #47043, Cell Signaling, Netherland), and human GYS1 (1:1000, #3886, Cell Signaling, Netherland) respectively. Equal loading was quantified using an antibody against GAPDH (1:1000, Cell Signaling). The TBS-T wash step was repeated before incubation with the HRP-conjugated antibody (raised against the primary antibody), for 1h at RTP (1:2000, Cell Signaling). Protein bands were visualized using a chemiluminescent detection kit (Advansta, Biozym, Germany) using iBright^TM^ Imaging System (Invitrogen). All experiments were performed in 3 or more cell cultures. Bands were quantified with ImageJ Software.

### Cellular glucose uptake

The fluorescent glucose analogue 2-(N-(7-nitrobenz-2-oxa-1,3-diazol-4-yl)amino)-2-deoxyglucose (2-NBD-glucose; Invitrogen, Darmstadt, Germany) was used to measure the relative uptake of glucose by flow cytometry. In each condition, cells were incubated with 2-NBD-glucose (30 μM) for 1 hour at 37 °C, subsequently washed twice in cold PBS and subjected to flow cytometry (BD Biosciences, Heidelberg, Germany) in fluorescence channel FL1. Data were analyzed using the FlowJo Software.

### Cellular glycogen content

At the end of the experiment, glycogen concentration was measured using a Glycogen Assay Kit (MAK016, Sigma, Germany), according to the manufacturer's protocol. Samples were measured using the Sunrise ELISA plate reader (Tecan, Germany)

### Statistics and data availability

The data are given as arithmetic means ± SEM, *n* denotes the number of independent biological experiments. The data were analyzed for significance using unpaired Student’s *t*-test using GraphPad Prism Software (CA, USA). Statistical significance was considered when *p*< 0.05. All relevant data are within the manuscript and its supporting information files.

## Results

**Fig 1:**
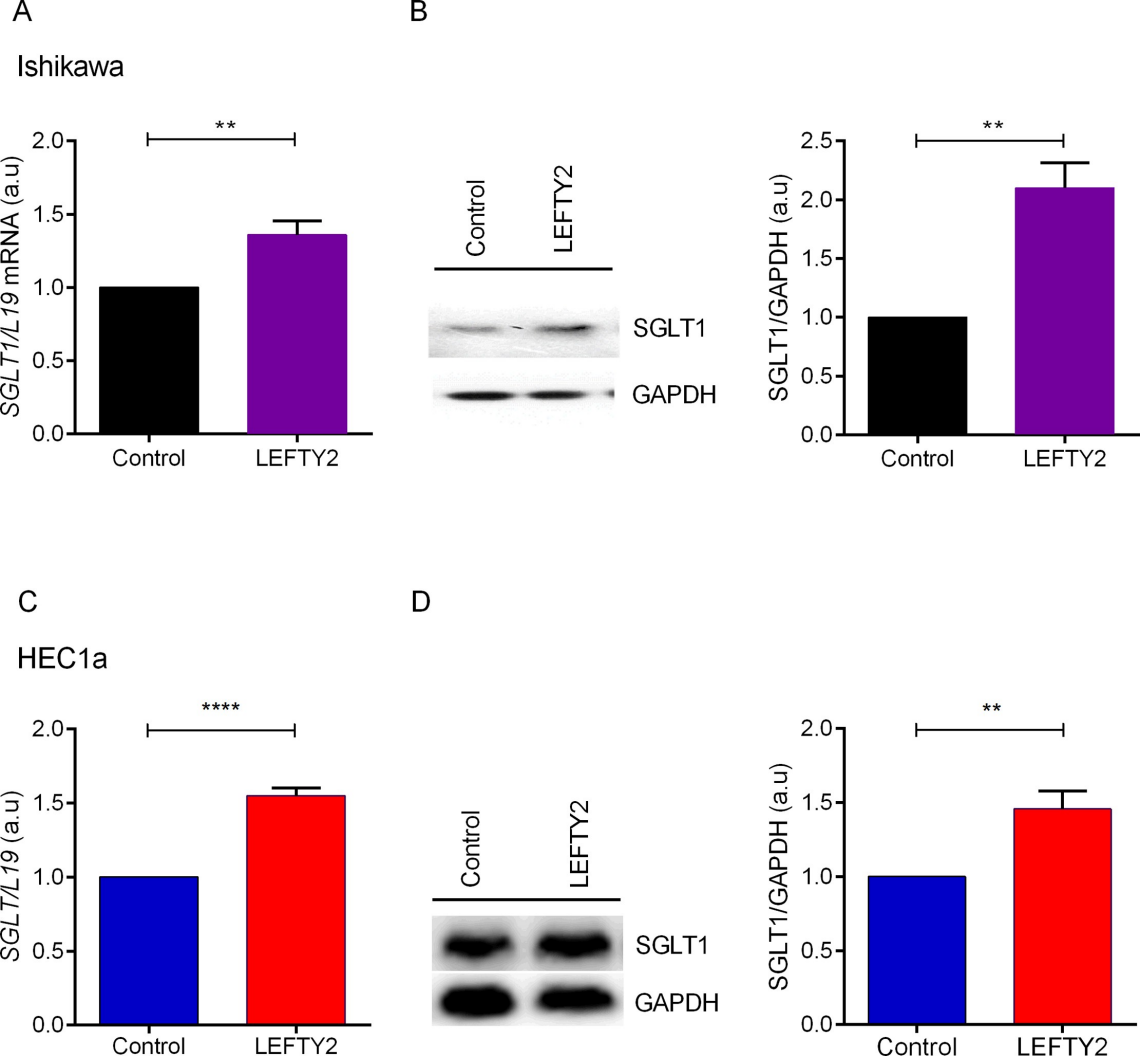
Effect of LEFTY2 on *SGLT1* transcript levels and protein abundance. **A**. Arithmetic means ± SEM (n = 4) of *SGLT1* transcript levels from Ishikawa cells without (black) or with treatment (purple) with 25 ng/ml LEFTY2 for 48 hours. *L19* was used as a housekeeping control. **B.** An original western blot of SGLT1 and GAPDH protein in whole cell lysates from Ishikawa cells without or with a 48 hours treatment with LEFTY2 (25 ng/ml). GAPDH was used as a loading control. Arithmetic means ± SEM (n = 4) of the SGLT1/GAPDH protein abundance ratios in cell lysate from Ishikawa cells without or following treatment with 25 ng/ml LEFTY2 (right side). **C.** Arithmetic means ± SEM (n = 5) of *SGLT1* transcript levels from HEC1a cells without (blue) or with treatment (red) with 25 ng/ml LEFTY2 for 48 hours. *L19* was used as a housekeeping control. **D.** An original western blot of SGLT1 and GAPDH protein in whole cell lysates from HEC1a cells without or with a 48 hours treatment with LEFTY2 (25 ng/ml). GAPDH was used as a loading control. Arithmetic means ± SEM (n = 7) of the SGLT1/GAPDH protein abundance ratios in cell lysate from HEC1a cells without or following treatment with 25 ng/ml LEFTY2 (right side). **(*p*<0.01) indicates statistically significant difference from untreated cells.

The present study addressed the effect of LEFTY2 on the Na^+^ coupled glucose transporter SGLT1 and glucose utilization in Ishikawa and HEC1a cells. In the first series of experiments the expression of SGLT1 was determined. Quantitative real-time PCR was utilized to determine the *SGLT1* transcript levels. Ishikawa cells and HEC1a cells remained untreated or were treated with LEFTY2 (25 ng/ml) for 48 hours. As shown in **Fig 1A**, the expression of *SGLT1* transcript levels in Ishikawa cells was significantly enhanced following LEFTY2 treatment. As illustrated in **Fig 1B**, according to Western blotting, treatment of Ishikawa cells with 25 ng/ml of LEFTY2 for 48 hours was followed by a marked and significant increase of SGLT1 protein abundance. These effects were also seen in parallel in HEC1a cancer cells (**Fig 1C & 1D**). However, LEFTY2 was unable to increase SGLT1 levels in benign endometrial cells. (**S1A Fig**).

**Fig 2:**
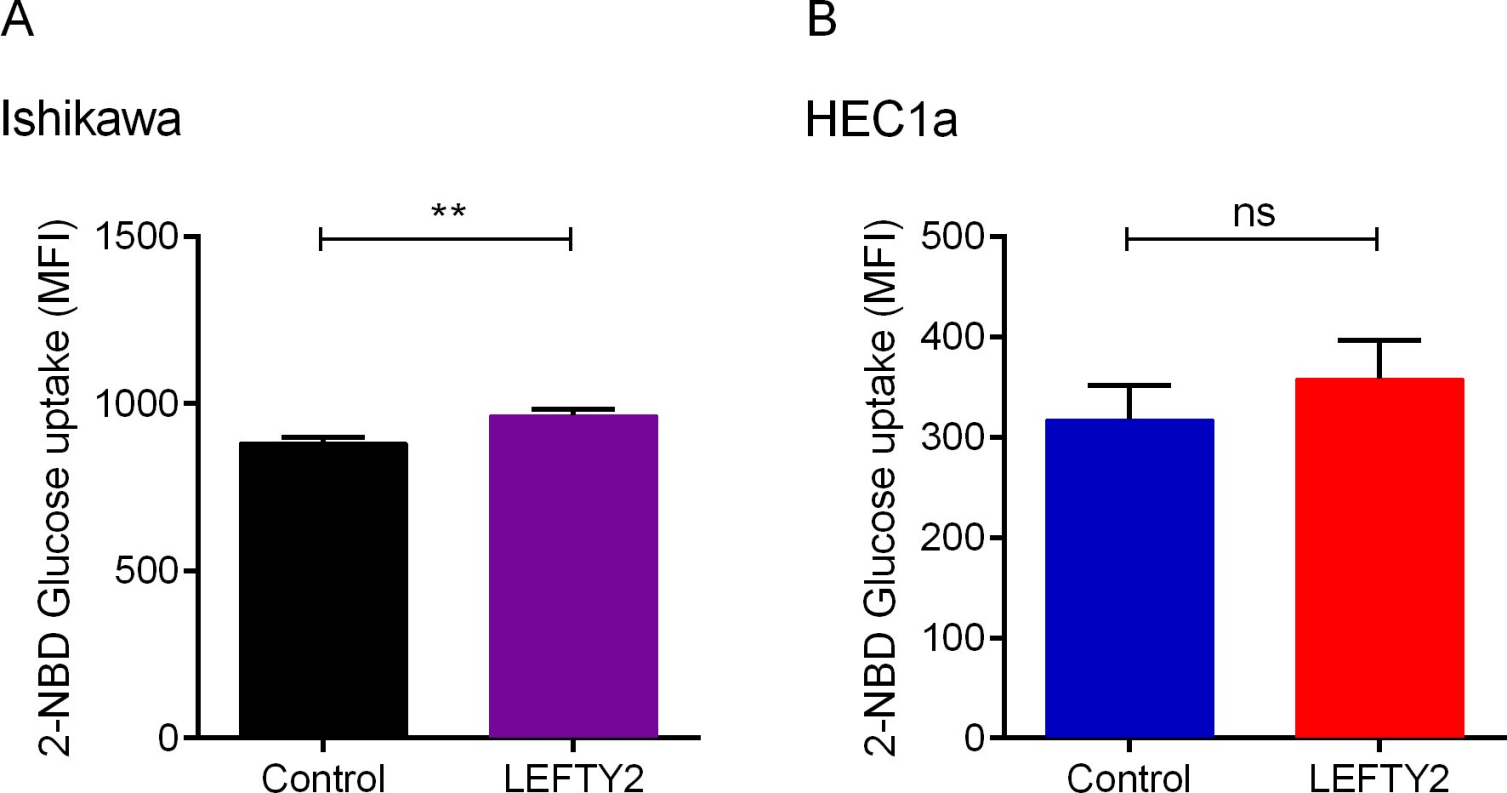
Effect of LEFTY2 on glucose uptake. Arithmetic means ± SEM of glucose uptake in (**A**) Ishikawa cells (n = 9) or (B) HEC1a cells (n = 6) untreated (control) or treated with 25ng/ml LEFTY2 for 48 hours and incubated with 2-NBDG (a fluorescent glucose analog) for 1 hour and analysis by flow cytometry. ** (*p*<0.01) indicates statistically significant difference from untreated cells) using Student’s t-test.

In the next set of experiments, we sought to quantify glucose transport. Ishikawa cells and HEC1a cells were treated with or without LEFTY2 (25 ng/ml) for 48 hours. One hour prior to the end of the experiment the cells were treated with a fluorescent glucose analog, 2-[*N*-(7-nitrobenz-2-oxa-1,3-diaxol-4-yl)amino]-2-deoxyglucose (2-NBDG). The uptake of the fluorescent substrate 2-NBDG was quantified using flow cytometry. As illustrated in **Fig 2**, treatment of Ishikawa cells and HEC1a cells with 25 ng/ml of LEFTY2 for 48 hours was followed by an increase of cellular 2-NBDG (glucose) uptake. However, it did not reach significance in HEC1a cells.

Next, in order to define the use of increased glucose uptake in endometrial cancer cells treated with LEFTY2, quantitative real-time PCR was utilized to determine *GYS1* transcript levels. Ishikawa cells or HEC1a cells remained untreated or were treated with LEFTY2 (25 ng/ml) for 48 hours. As shown in **S2A & S2B Fig**, the transcription of *GYS1* was significantly enhanced following LEFTY2 treatment in both cell lines.

**Fig 3:**
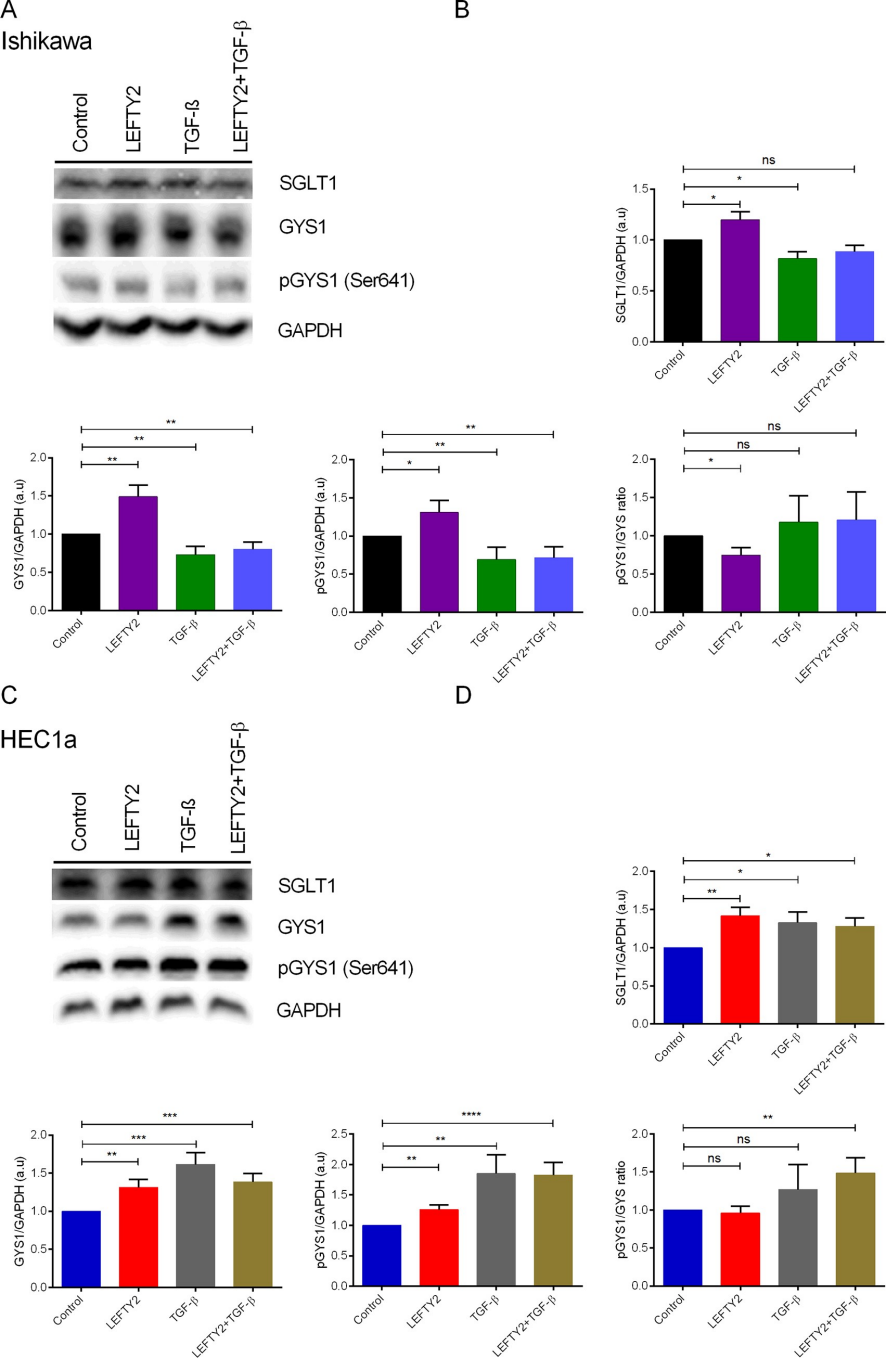
Effect of TGF-β on SGLT, GYS1 and on pGYS1 (Ser641) protein levels. **A.** Original western blots of SGLT1, GYS1, pGYS1 (Ser641), and GAPDH protein in whole cell lysates from Ishikawa cells without or with a 48 hours treatment with LEFTY2 (25 ng/ml) or with TGF-β (10 ng/ml) or in combination. GAPDH was used as a loading control. **B.** Arithmetic means ± SEM (n = 10) of SGLT1/GAPDH, GYS1/GAPDH (n = 9) and pGYS1 (Ser641)/GAPDH protein (n = 6) abundance ratios in cell lysates. **C.** Original western blots of SGLT1, GYS1, pGYS1 (Ser641), and GAPDH protein in whole cell lysates from HEC1a cells without or with a 48 hours treatment with LEFTY2 (25 ng/ml) or with TGF-β (ng/ml) or together. GAPDH was used as a loading control. **D.** Arithmetic means ± SEM (n = 10) of SGLT1/GAPDH, GYS1/GAPDH (n = 5) and pGYS1 (Ser641)/GAPDH protein (n = 4) abundance ratios in cell lysates. *(*p*<0.05), **(*p*<0.01) indicates statistically significant difference from untreated cells.

Glycogen, a polysaccharide of glucose, serves as energy storage. GYS1 catalyzes the rate-limiting step of glycogen biosynthesis. GYS1 is inactivated by phosphorylation at several sites, and activation occurs by dephosphorylation [47]. To investigate GYS1 activity at protein level, Ishikawa cells or HEC1a cells were treated with 25 ng/ml of LEFTY2 for 48 hours and whole cell lysates were subjected to Western blotting. As illustrated in **Fig 3**, treatment with LEFTY2 resulted in an increase of total GYS1 protein abundance in both Ishikawa (**Fig 3A & 3B**) and HEC1a (**Fig 3C & 3D**) cells. Further, treatment with LEFTY2 resulted in a decrease of phosphorylated GYS1 (Ser641)/ GYS1 ratio when compared with the control suggesting active glycogen synthesis in Ishikawa cells. HEC1a cells showed no apparent change. No effect was seen on benign endometrial cells (**S1B Fig).**

LEFTY2 has been described as a TGF-β antagonist and as a tumor supressor. We investigated whether TGF-β can inhibit glycogen synthesis induced by LEFTY2. We treated Ishikawa and HEC1a cells without or with LEFTY2 (25 ng/ml) or with TGF-β (10 ng/ml) for 48 hours. To test any antagonistic effects the cells were pre-treated with TGF-β (10 ng/ml) for 24 hours and then treated with LEFTY2 and TGF-β for a further 48 hours. Here, we show that treatment with TGF-β or in combination LEFTY2 decreased SGLT1, GYS and pGYS1 (Ser641) levels in both Ishikawa (**Fig 3A & 3B; S2A Fig**) and HEC1a cells (**Fig 3C & 3D; S2A Fig**).

**Fig 4:**
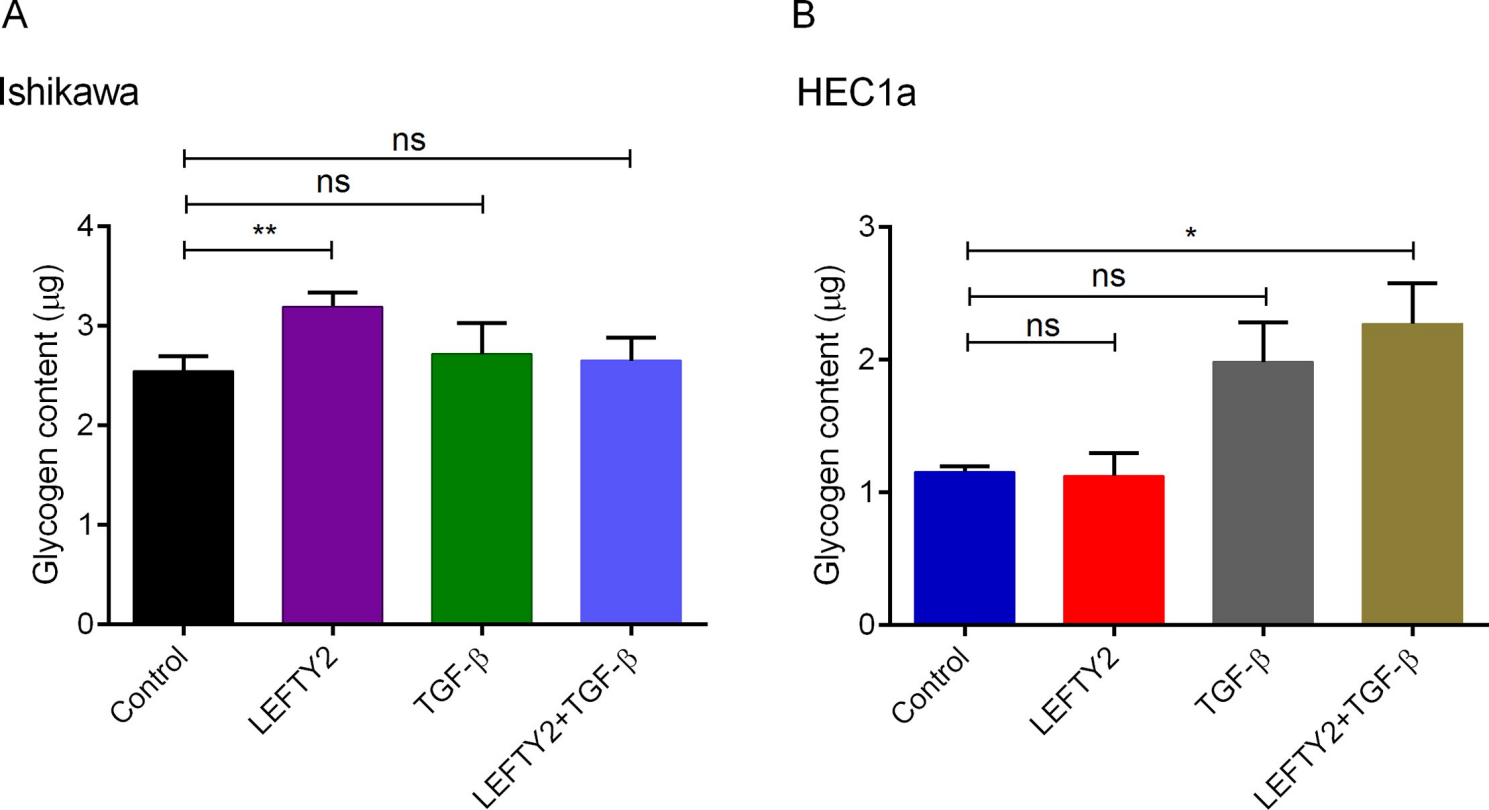
Effect of LEFTY2 on glycogen levels. Arithmetic means ± SEM glycogen content in (A) Ishikawa cells (n = 7) or (B) HEC1a cells (n = 4). Untreated (control) or treated with 25ng/ml LEFTY2 for 48 hours or with TGF-β (10 ng/ml) alone or in combination. *(*p*<0.05), ** (*p*<0.01) indicates statistically significant difference from untreated cells (control).

To investigate if indeed LEFTY2 stimulates glycogen accumulation, we treated Ishikawa and HEC1a cells with or without LEFTY2 (25 ng/ml) for 48 hours and measured glycogen content using an ELISA based method. Here, we show that a 48 hours treatment with LEFTY2 significantly increased cellular glycogen content only in Ishikawa cells (**Fig 4A & 4B**). Intresetingly, treatment with TGF-β (10 ng/ml) alone or in combination negated the LEFTY2-driven glycogen accumulation in Ishikawa cells. No effect was seen on HEC1a cells.

## Discussion

The present observations reveal a novel function of LEFTY2 in the regulation of SGLT1. According to our previous observations [31], LEFTY2 is a potent inhibitor of glycolytic flux in Ishikawa cells. Further, LEFTY2 decreased lactate concentration in the supernatant of Ishikawa cells [31]. This negative effect of LEFTY2 on glycolysis was explained by a marked cytosolic acidification, which was in part due to an inhibitory effect of LEFTY2 on Na^+^/H^+^ exchanger activity [31]. Glycolytic flux is highly sensitive to cytosolic pH and is disrupted by cytosolic acidification [32].

The present observations confirm that LEFTY2 increased transcript levels and protein abundance of *SGLT*1. Moreover, LEFTY2 increased the cellular uptake of the glucose carrier substrate 2-NBDG, an observation again pointing to enhanced carrier activity. It is tempting to speculate that the simultaneous increase of cellular glucose uptake (by SGLT1 transporter) and inhibition of glycolysis served to foster the cellular formation of glycogen, an endometrial cancer-specific function. Notably, glycogen which is stored in the endometrium and considered to serve as an important energy source in early pregnancy [48]. Recently it has been shown that loss of SGLT1 impairs adequate endometrial glycogen stores for pregnancy and disruption of this histotrophic pathway leads to adverse pregnancy outcome and miscarriage in both humans and mice [44]. GYS1 activation occurs by dephosphorylation, thus increasing the cells capacity for glycogen storage [47]. Our data reveal that LEFTY2 indeed increased cellular glycogen abundance via GYS1 activation. Our additional experiments have showed no role of LEFTY2 on building glycogen stores and thus energy reserves in benign endometrium. Moreover, using HEC1a cells we also did not see an increase in glycogen accumulation this maybe in part due to a cell-line specific effect, further experiments are warranted to dicepher this. These observations reveal completely novel insight into the effect of LEFTY2 on glycolysis in endometrial cancer cells. We did not find that LEFTY2 can antagnosise TGF-β signaling. The combined stimulation of cellular glucose uptake and inhibition of glycolysis may serve to boost the cellular formation of glycogen. In this respect, the utilization of SGLT1 may be particularly valuable, as it is capable of cellular glucose uptake against a steep chemical gradient and is able to accomplish cellular glucose uptake at low extracellular and high intracellular glucose concentrations [49]. Further, our data supports the hypothesis that TGF-β could act as a tumor suppressor potentially by reducing glycogen accumulation in endometrial cancer cells. Further work is needed to confirm this conjecture.

Tumor cells are dependent on delivery of glucose to cover their excessive demand for this substrate [50]. Even in the presence of glucose, tumor cells degrade glucose to lactate and thus utilize only a small fraction of the energy, which could be generated by oxidative degradation of glucose [31, 51]. At least, in some tumor cells, the uptake of glucose with facilitative glucose carriers alone presumably fails to supply sufficient amounts of glucose. SGLT1 couples the uphill transport of glucose to Na^+^ entry down its electrochemical potential gradient across the plasma membrane and is thus able to accomplish cellular glucose uptake when extracellular glucose concentrations are below those within the cell [17, 38].

Growing evidence suggests that those with type-2 diabetes are at elevated risk for cancer [52, 53]. Several carcinogenic risk factors involving the pathophysiology of type-2 diabetes, such as *obesity*, play a significant role in increasing endometrial cancer risk [52]. SGLT inhibitor-drugs developed for diabetes may be beneficial in treating cancers, either alone or in combination with other anti-cancer treatments. It is well established that increased glucose uptake and aerobic glycolysis are landmark signals of cancer cells (both are targets for cancer therapy) [11]. It is therefore, a huge clinical challenge to selectively inhibit glucose uptake in tumors, without disturbing the normal physiology of non-affected organs (heart, muscle, brain, etc.). However, in those tumors where glucose uptake occurs through SGLTs, it may be possible to significantly reduce glucose uptake and cell growth by inhibiting SGLT activity, though further studies are required to support this hypothesis [39, 40]. Moreover, additional experiments may uncover the coincidence of stimulated cellular glucose uptake and inhibition of glycolysis as a strategy to boost glycogen formation in other cell types.

In conclusion, the present study demonstrates a novel role of LEFTY2 in up-regulating transcript levels and protein abundance of the Na^+^ coupled glucose transporter SGLT1, thus stimulating cellular glucose uptake and cellular formation of glycogen in endometrial cancer cells.

## Supporting information

S1 Fig No effect of LEFTY2 on benign endometrial cells. **A.** Arithmetic means ± SEM (n = 4) of *SGLT1* and (n = 5) *GYS1* transcript levels from benign endometrial cells without or with treatment with 25 ng/ml LEFTY2 for 48 hours. *L19* was used as a housekeeping control. **B.** An original Western blot of SGLT1, GYS1, pGYS1 (Ser641), and GAPDH protein in whole cell lysates from benign endometrial cells without or with a 48 hours treatment with LEFTY2 (25 ng/ml). GAPDH was used as a loading control. Arithmetic means ± SEM of SGLT1/GAPDH (n = 6), GYS1/GAPDH (n = 7) and pGYS1 (Ser641)/GAPDH (n = 7) protein abundance ratios in cell lysate without or following treatment with 25 ng/ml LEFTY2.
(TIF)

S2 Fig Co treatment with TGF-β reduces SGLT1 and GYS1 transcript levels in Ishikawa and HEC1a cells. **A.** Ishikawa cells or **B.** HEC1a cells were treated 48 hours treatment with LEFTY2 (25 ng/ml) or with TGF-β (10 ng/ml) or in combination. Control cells remained untreated. Arithmetic means ± SEM (n = 5) of *SGLT1* and *GYS1* transcript. *L19* was used as a housekeeping control. *(*p*<0.05) and **(*p*<0.01) indicates statistically significant difference from untreated cells (control).
(TIF)

S1 Data (XLSX)

S2 Data (XLSX)
